# Internal Gas-Assisted Mold Temperature Control for Improving the Filling Ability of Polyamide 6 + 30% Glass Fiber in the Micro-Injection Molding Process

**DOI:** 10.3390/polym14112218

**Published:** 2022-05-30

**Authors:** Tran Minh The Uyen, Thanh Trung Do, Pham Son Minh

**Affiliations:** Faculty of Mechanical Engineering, HCMC University of Technology and Education, Ho Chi Minh City 71307, Vietnam; uyentmt@hcmute.edu.vn (T.M.T.U.); trungdt@hcmute.edu.vn (T.T.D.)

**Keywords:** injection molding, mold heating, dynamic mold temperature control, internal gas-assisted mold temperature control, micro-injection molding product, melt flow length

## Abstract

In micro-injection molding, the plastic filling in the cavity is limited by the frozen layer due to the rapid cooling of the hot melt when it comes into contact with the surface of the cavity at a lower temperature. This problem is more serious with composite materials, which have a higher viscosity than pure materials. Moreover, this issue is also more serious with composite materials that have a higher weight percentage of glass filer. In this article, a pre-heating step with the internal gas heating method was used to heat the cavity surface to a high temperature before the filling step to reduce the frozen layer and to improve the filling ability of the composite material (polyamide 6 + 30% glass fiber) in the micro-injection molding process. To heat the cavity surface, an internal gas-assisted mold temperature control (In-GMTC) system was used with a pulsed cooling system. We assessed different mold insert thicknesses (t) and gaps between the gas gate and the heating surface (G) to achieve rapid mold surface temperature control. The heating process was observed using an infrared camera, and the temperature distribution and the heating rate were analyzed. Thereafter, along with the local temperature control, the In-GMTC was used for the micro-injection molding cycle. The results show that, with a gas temperature of 300 °C and a gas gap of 3.5 mm, the heating rate reached 8.6 °C/s. The In-GMTC was also applied to the micro-injection molding process with a part thickness of 0.2 mm. It was shown that the melt flow length had to reach 24 mm to fill the cavity completely. The results show that the filling ability of the composite material increased from 65.4% to 100% with local heating at the melt inlet area when the gas temperature rose from 200 to 400 °C with a 20 s heating cycle.

## 1. Introduction

Nowadays, in the injection molding field, controlling the temperature of the mold is a key point in plastic processing. In common processes, the cooling time is reduced by decreasing the mold surface temperature in the molding cycle, but this does not enhance the surface detail quality. In order to improve the detail quality, it is necessary to increase the mold surface temperature. However, this solution increases the cooling and cycle times. Therefore, the most important objective of current research is to increase the mold surface temperature while maintaining a short cycle time. In the molding-cycle filling step, the mold temperature directly impacts the filling ability of the plastic material, especially with the composite plastic. The low mold temperature tends to negatively affect the filling step. This influence is more important in the micro-injection molding process, in which the melt flow length must be increased in the product’s micro-features. Because micro-injection molds are low cost and have the potential for high-volume production, they are used to manufacture a variety of polymer components in the injection molding field [1,2,3,4,5,6,7,8]. Therefore, the most common applications of micro-injection molding are in micro-devices [9,10], optical structures [11,12,13,14], and micro-fluidic devices [15,16,17]. Other molded micro-optical components, including optical gratings; optical switches; waveguides; and a variety of molded micro-fluidic devices, such as micro-pumps, capillary analysis systems, and lab-on-a-chip applications, are now being manufactured.

At present, various types of dynamic mold temperature control (DMTC) have been studied, which eliminates the frozen layer by applying a high mold temperature at the cavity surface in the filling stage and maintains the plate, which has a low temperature in the cooling stage. This makes it possible to maintain a high mold temperature during filling while reducing the mold temperature below the ejection temperature during post-filling without significantly increasing the cycle time or energy consumption. Therefore, a higher mold temperature during injection is required to accommodate the micro-part and reduce the injection pressure to improve the filling ability in the injection molding cycle.

To maintain a high mold temperature in the filling step, the simplest method is to use the hot fluid to raise the mold temperature. In this method, hot water at temperatures as high as 90 °C to 100 °C flows into the channels of the mold plate and increases the plate temperature. This is the most inexpensive way to increase the mold temperature. Another way for assisting high mold temperature control is local mold heating with an electric heater [18,19,20,21]. This method, however, requires additional design and tooling costs. Furthermore, electrical heating is typically used as an auxiliary heating method and is limited to temperature increases of a few 10 s of degrees centigrade. In general, these heating methods not only increase the temperature of the cavity surface, but the cavity plate is also heated. Therefore, the heating energy is wasted, and the cooling time increases because the removing of thermal energy in one cycle is large.

These issues can be addressed by directly heating the cavity surface. There are several methods that can support a high heating rate and good predictability without heating the entire cavity volume, for example, induction heating [22,23,24,25], high-frequency proximity heating [26,27], and gas-assisted mold temperature control (GMTC) [28,29,30,31,32]. The induction heating method has a very fast heating rate, which is a great advantage. However, the temperature distribution is still a challenge to control. In particular, induction heating is only used on high permeability/quality steel molds.

On the other hand, to prevent the mold from overheating as a result of induction heating, especially the edge area of the mold plate, hot gas was suggested as a heating source for increasing the mold surface temperature. Gas heating does not have the same heating rate as induction heating, but this method can be used for any mold material. Furthermore, because of the heat convection between the hot gas and the mold surface, gas heating has the potential to protect the mold from overheating. In previous research, the gas heating structure was assembled into the mold to determine heating efficiency, which included the heating rate and temperature distribution [30,31,32]. The results of the tests were positive. However, during these experiments, difficulty in incorporating the gas heating system into the mold construction and the significant loss of thermal energy as air was transferred from the heating source to the heating surface were reported. For solving the trouble in this design, in this research, the heating system was redesigned with the heating position at the back side of the cavity insert. The detail of this design is described in the next part. With this design, the hot gas was controlled easier by a closed volume, and thus the heating efficiency was improved.

## 2. Experimental Method

**Figure 1:**
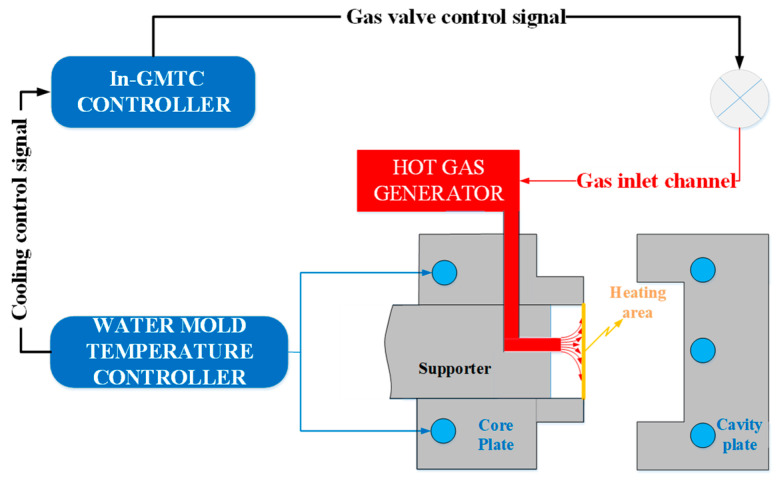
The In-GMTC system.

In this research, the In-GMTC system is made up of two main components, as shown in Figure 1. They are a water mold temperature controller and a hot-gas generator system (including an air compressor, an air drier, a digital volumetric flow controller, and a high-efficiency gas heater). The system’s construction allows it to quickly heat and cool the mold surface during injection molding. The purpose of mold temperature control is to increase the mold surface temperature to the target temperature, before the mold is filled with melt, and then cool the melt to the ejection temperature. With this design, as compared with previous research [31,32,33,34], the heating source is closer to the heating area, and thermal loss is reduced. In addition, the heating stage occurs in a closed volume, and thus the issue of fluid dispersal is solved.

In this research, the function of the high-power hot-gas generator system is to support a heat source, providing a flow of hot air of up to 400 °C with an inlet gas pressure of up to 7 bars, which is the same as in previous research concerning gas-assisted mold temperature control. A mold temperature controller was used to provide water at a defined temperature in order to cool the mold after the filling process and to warm the mold to the initial temperature at the beginning of each experiment for the cooling system. To maintain the initial mold temperature, the water flowed during the molding cycle. Therefore, with the design shown in Figure 1, the water mold temperature controller is the same as in the traditional injection molding process. As compared with other mold heating methods such as external induction heating [23] or external gas-assisted mold temperature control [29], this has the advantage of using an In-GMTC.

**Figure 2:**
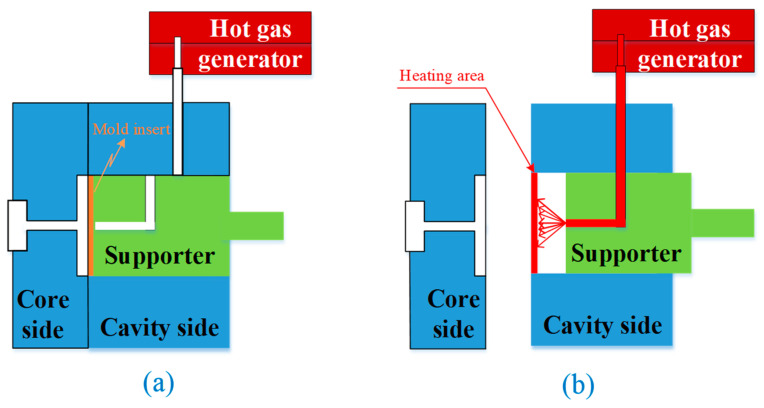
Mold position in the heating stage of the In-GMTC process. (**a**) Molding position. (**b**) Heating position.

In the heating step, the hot gas is used as a heating source to increase the insert temperature of the injection mold. First, when the two halves of the mold move into the closed position in preparation for the filling step (Figure 2a), the supporter moves back to the heating position, as shown in Figure 2b. Second, the air source, dried by the air dryer, moves through the gas generator into the heating position. Then, the hot air is in direct contact with the insert surface. This hot gas heats the insert to the target temperature. Third, the supporter is moved into contact with the insert plate when the mold reaches the target temperature. Thereafter, the mold cycle continues as the melt filling process starts. In this method, the hot gas does not come into direct contact with the cavity surface. Therefore, the heating efficiency is impacted by the insert thickness. A thicker insert allows for a lower heating rate. However, an insert that is too thin negatively affects the rigidity of the insert. In addition, when the hot gas heats the insert, the gap between the supporter and the insert impacts the flow of hot air. If the gap is the too large or too thin, the heating rate and the temperature uniformity are negatively affected.

Although the In-GMTC has various advantages—such as a high heating rate, the overheating issues being addressed, and it being able to be applied for different products—applying this method in the molding cycle still has many issues. Therefore, in this research, an internal gas-assisted mold temperature control (In-GMTC) was established to achieve rapid mold surface temperature control, using different mold insert thicknesses (t) and gaps between gas gate and heating surface (G). The effect of the heating parameters on the insert plate, such as heating efficiency and temperature distribution uniformity, was studied through a series of systematic tests. Internal air-assisted heating for mold surface temperature adjustment during the injection process to improve the melt flow length was assessed using this system on a mold for a micro-product with a filling material of polyamide 6 + 30% glass fiber. To estimate the improvement in the filling step in the micro-injection molding product, the melt flow length and the filling percentage values of the composite material were measured.

**Figure 3:**
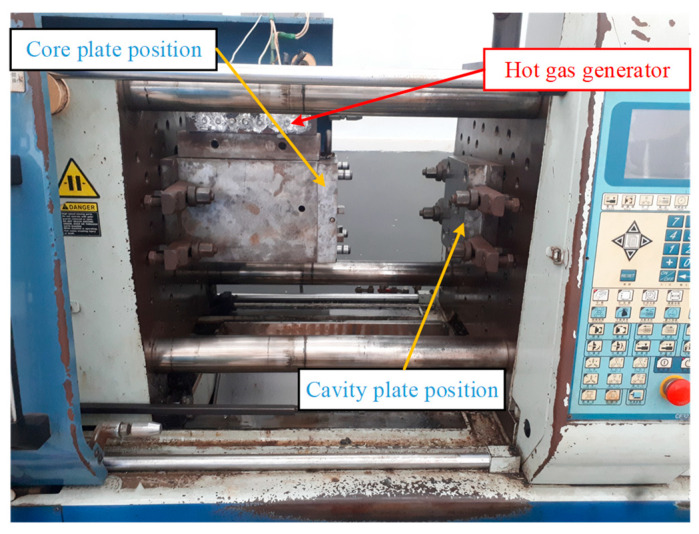
The experiment model for the In-GMTC.

**Figure 4:**
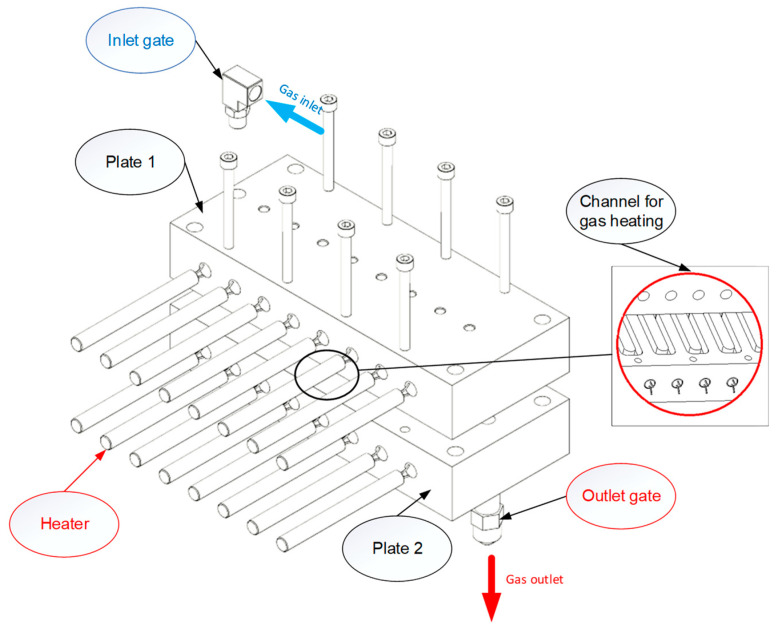
The hot-gas generator.

**Figure 5:**
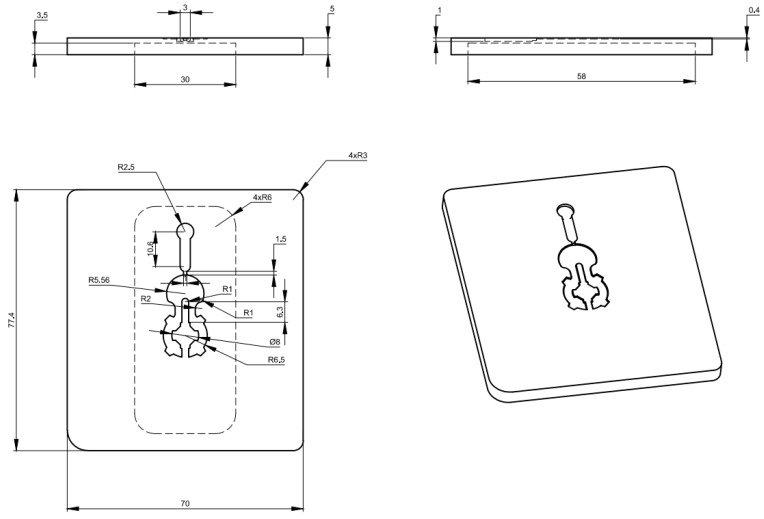
Cavity dimensions.

**Figure 6:**
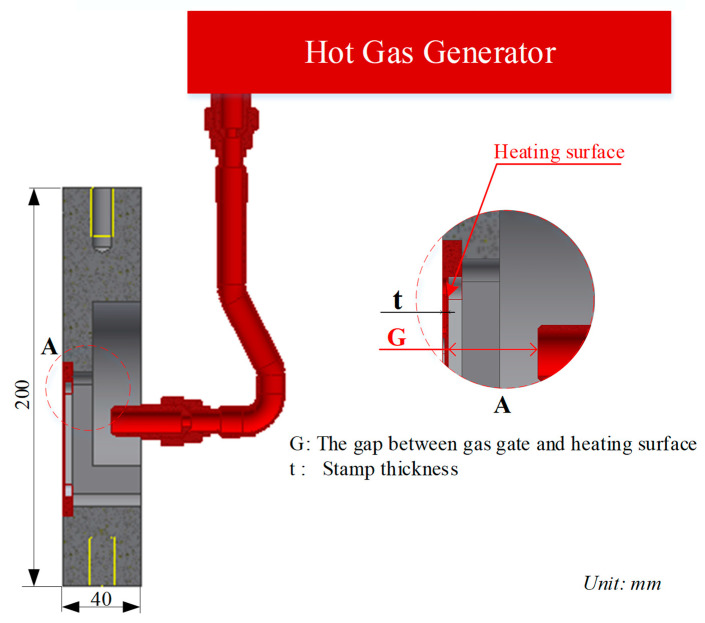
Mold structure at the heating position.

In this paper, Figure 3 depicts the position of the In-GMTC and the mold plate on the injection molding machine during application. Figure 4 depicts the hot-gas generator with dimensions of 240 × 100 × 80 mm^3^. The gas channel was cut with a width of 5 mm and a depth of 10 mm inside the gas drier. In this study, the heating area of the mold cavity was inserted using an insert measuring 77.4 × 70 mm^2^. The cavity’s dimensions are shown in Figure 5. Figure 6 depicts the position of all systems in the heating step in both the simulation and experiment.

## 3. Simulation Method

**Figure 7:**
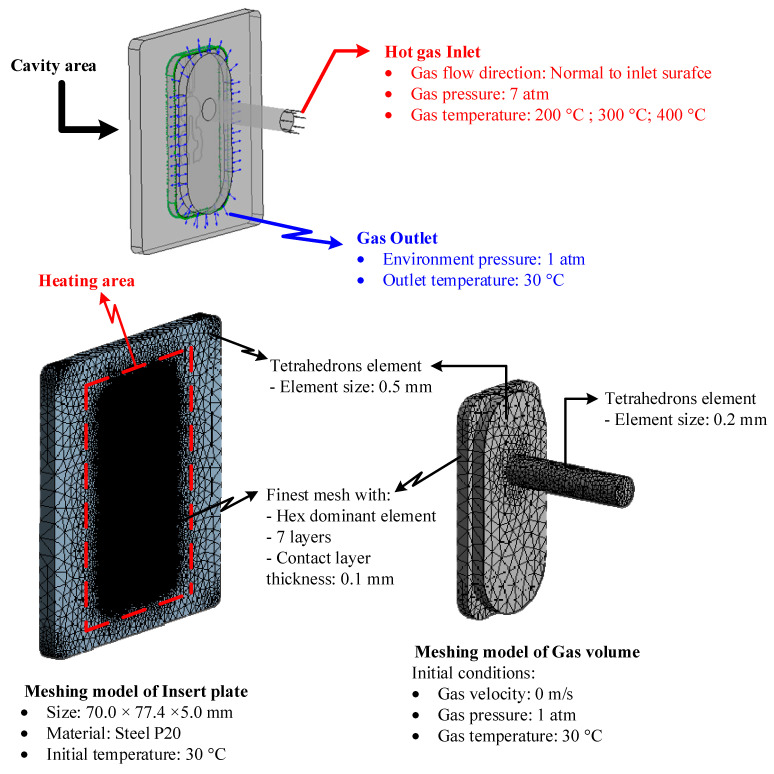
Simulation model and boundary conditions.

In the In-GMTC, there are many parameters that affect the heating process, but the stamp thickness is the most important parameter that directly influences the heating process and the filling stage of the molding cycle. Therefore, in order to study the temperature distribution of the heating area, the simulation model was built as in the experiment. In other studies, the simulation model only included two volumes: the stamp volume and the air volume. In reality, when the stamp is inserted into the mold, there is a small air gap between the stamp and the mold, and this gap plays the role of a short-term insulating layer. The geometry view, meshing model, and boundary condition of the system are shown in Figure 7. The material properties of air and steel from the simulation are shown in Table 1. In the meshing model, the stamp was meshed with a hex dominant element, which had seven layers in the thickness direction in order to enhance the simulation accuracy, and the air volume was meshed with a tetrahedron element with a smaller element size at the hot-gas inlet location. The ANSYS software (2018, ANSYS, Inc., Ho Chi Minh City, Vietnam) was used to simulate the heating process using the same experimental parameters.

**Figure 8:**
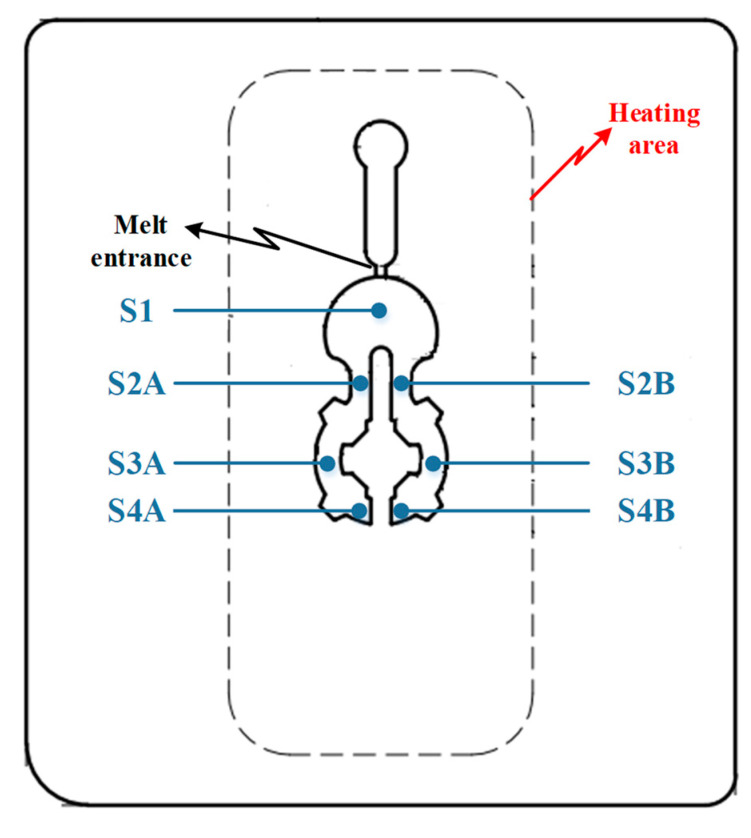
The sensor locations.

The inserts and the temperature measuring points, as shown in Figure 8, were used for the experiment to observe the influence of the In-GMTC on the injection molding process. With the normal injection molding process, the product thickness is 0.2 mm, which is a kind of micro-injection molding and often suffers from short shot when the injection pressure is low; however, with too high an injection pressure, the flash trouble often occurs. Therefore, as a result of its ability to control the mold temperature, the In-GMTC was used for this molding process in order to assess the product quality enhancement when the injection molding process was operated at a medium injection pressure. The plastic material PA6 + 30%GF from LANXESS AG, Cologne, Germany, was used for the molding process in this study, and the molding parameters were maintained at a constant for all testing cases, as shown in Table 2. In this experiment, a SW-120B (Shine Well Machinery Co., Ltd. Taichung City, Taiwan) was used.

## 4. Results and Discussions

### 4.1. Effect of Stamp Thickness and Gas Gap on the Heating Process

**Figure 9:**
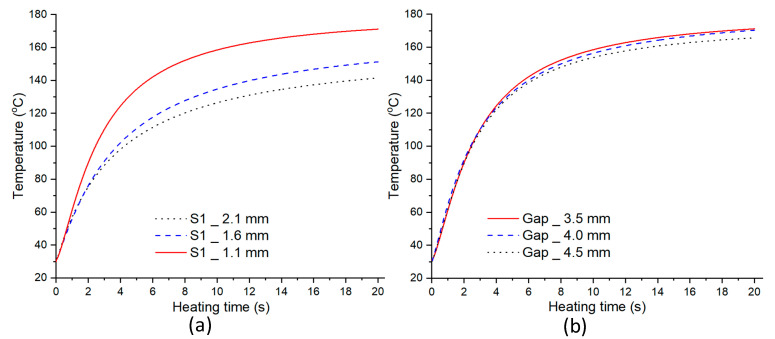
Temperature comparison at the center of the heating area (Point S1) in the simulation at 300 °C with different stamp thicknesses (**a**) and gas gap lengths (**b**).

In other studies, a stamped insert was frequently used to increase the heating efficiency when using the mold surface heating method. The stamp thickness is one of the most important parameters in mold design, according to the findings of these studies. Thus, inserts with a size of 77.4 × 70 mm^2^ were inserted into the cavity in this study to estimate the heating capacity of the In-GMTC, and the heating process was performed with a hot-gas temperature of 300 °C and a distance of 3.5 mm between the gas gate and the heating surface. Figure 9 depicts the variation in mold temperature for a heating time of 20 s. It can be seen that the In-GMTC can heat the plate to over 172 °C for an initial mold temperature of 30 °C. This demonstrates that the In-GMTC can heat at a rate of 8.6 °C/s with a change of stamp thickness from 1.1 to 2.1 mm and a change of gas gap length from 3.5 to 4.5 mm. This temperature is higher than all common plastic material glass transition temperatures. In our previous research concerning gas heating for the injection molding process, the hot gas was sprayed directly to the cavity surface for a heating area of 58 × 30 mm^2^, a gas flow rate of 500 L/min, and a gas temperature of 400 °C [28], and the maximum speed of the heating was only about 2.2 °C/s. This means that the design of the In-GMTC has a significant advantage in terms of heating efficiency on the surface of the mold.

**Figure 10:**
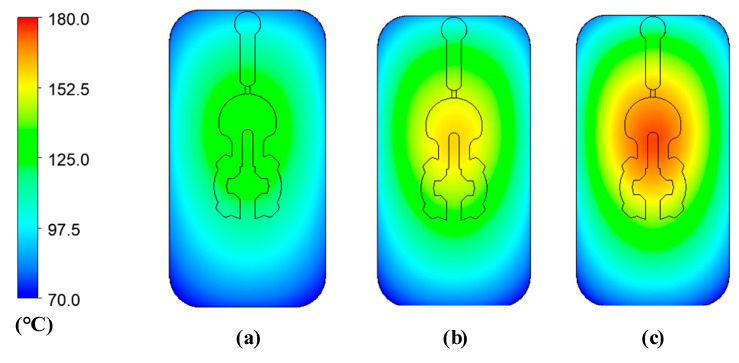
Temperature distribution of the molding area with an initial temperature of 30 °C; a gas temperature of 300 °C; a heating time of 20 s; and a stamp thickness of (**a**) 2.1 mm, (**b**) 1.6 mm, and (**c**) 1.1 mm.

**Figure 11:**
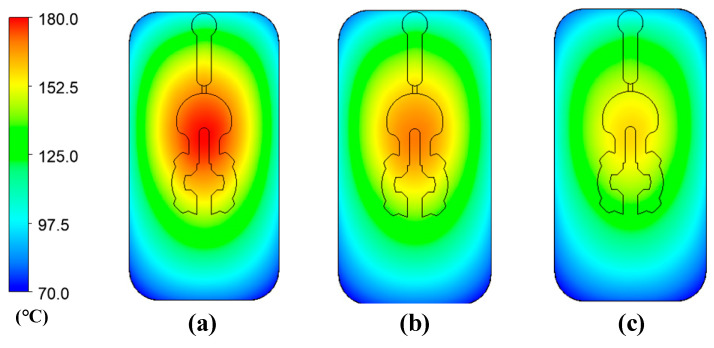
Temperature distribution of the molding area with an initial temperature of 30 °C; a gas temperature of 300 °C; a heating time of 20 s; and a gas gap length of (**a**) 3.5 mm, (**b**) 4.0 mm, and (**c**) 4.5 mm.

**Figure 12:**
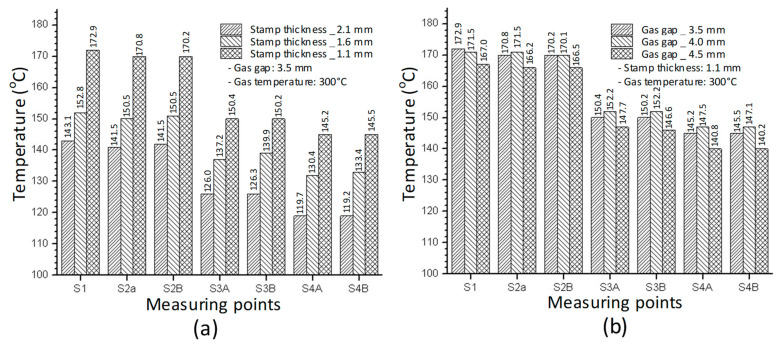
Temperature on the sensors in the simulation with different stamp thicknesses (**a**) and gas gap lengths (**b**).

Figure 10 and Figure 11 depict the temperature distribution simulation results with the change in stamp thickness and gas gap length. Figure 12 illustrates the temperature at the end of the heating stage at various sensor points. The maximum temperature was found at the top of the stamp, closest to the hot-gas gate, while the lowest temperature was found at the bottom of the stamp. This distribution is superior to previous GMTC studies [28,30,31,32], which frequently reported temperature imbalances between the two sides of the cavity region. Furthermore, when compared to induction heating, the In-GMTC could overcome the problem of low temperature in the heating area’s core [33,34], resulting in a superior application for actual molding products. These data also demonstrate that when the stamp thickness was 1.1 mm and gas gap length was 3.5, the highest temperature of 172 °C was reached. The capacity of these plates to transport heat might explain this increase. Thermal energy concentrates more so at the center of a plate that is thicker; however, when the stamp thickness decreases, it is more complicated for the thermal energy to move to the lower temperature area. These findings also show that with a heating duration of 20 s, the temperature of the stamp ranged from over 140 °C to over 170 °C for all types of stamp thickness. This indicates that this temperature range is ideal for injection molding since it is high enough to meet the glass transition temperature of almost every plastic material, yet the maximum temperature is not high enough to induce plastic deterioration. In addition, the heating rate is extremely high in the first 5 s; after that, the heating rate reduced. This result could be explained by the heat transfer from the gate heating area to the outside of the insert plate. At the beginning, the thermal energy was focused at the hot gate, and the temperature at the hot gate increased quickly. However, this thermal energy quickly transferred to the side area of the insert plate, so the temperature rate of increase at the hot gate slowed. In addition, at the beginning, the insert temperature was low, so the heat transfer from hot gas to the insert plate was facilitated. After that, the insert temperature increased, the heat transfer became harder, and the thermal energy absorbed by the insert was lower, which is the main reason for the reduction in the heating rate. Figure 9b shows the influence of the gap between the insert plate and the hot gate on the maximum temperature of the insert plate. In this simulation, the gap varied from 3.5 to 4.0 mm. The results show that this influence was not as clear as that of the insert thickness, as shown in Figure 9a. This was due to the fact that the hot gas was sprayed into a closed volume, so the thermal energy was focused on this volume, and the heating process was more stable. This result demonstrates an advantage of this heating method as compared with the external gas-assisted mold temperature control, which is strongly influenced by the gap between the hot gate and the heating surface [29]. However, by observing the temperature distribution in Figure 11, the influence of the gas gap was quite clear. This distribution clearly shows that, with the gap of 3.5 mm, the high temperature was focused at the hot gate, as compared with the 4.5 mm case. This means that with the larger gap, there was better temperature uniformity, but with a lower heating rate. In addition, the temperature distribution of all cases is very symmetrical, so the temperatures between the mirror points (S2A and S2B; S3A and S3B; S4A and S4B) are the same as in Figure 12.

**Figure 13:**
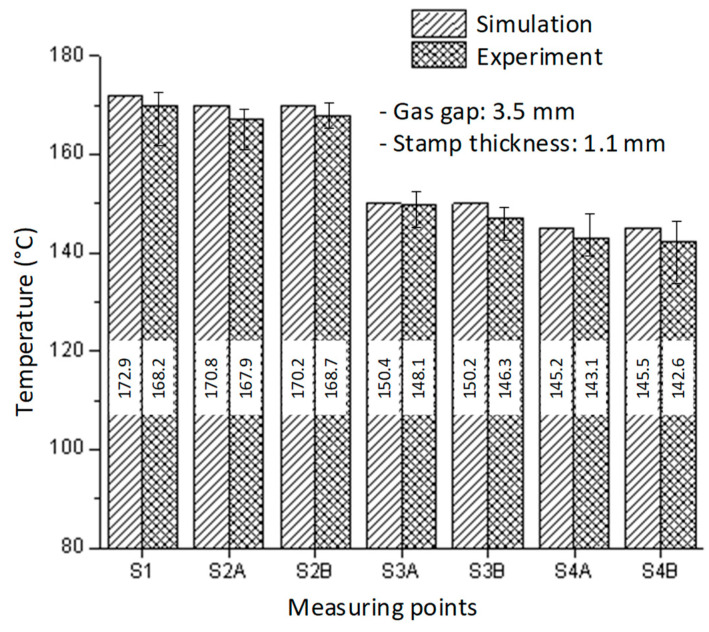
The comparison of temperatures in the simulation and experiment with a stamp thickness of 1.1 mm and gas gap of 3.5 mm.

In order to assess the simulation accuracy, the case of a 1.1 mm insert thickness and a 3.5 mm gas gap was selected for the experiment. The experiment was carried out with the identical boundary conditions as in the simulation to ensure that the simulation results were accurate. The experiment was repeated 10 times for each scenario, and the average value is used in this paper. The temperatures at the measuring points were then measured and compared to the modeling results, as shown in Figure 13. The temperature difference between the simulation and experiment was less than 5 °C. This disparity was due to the sensor’s latency in measuring the temperature. In this situation, thermal energy transfers swiftly from the high temperature to the lower temperature. However, in general, this conclusion indicates that the simulation and experimental results are in excellent accord.

### 4.2. Effect of the Inlet Temperature on the Heating Process

**Figure 14:**
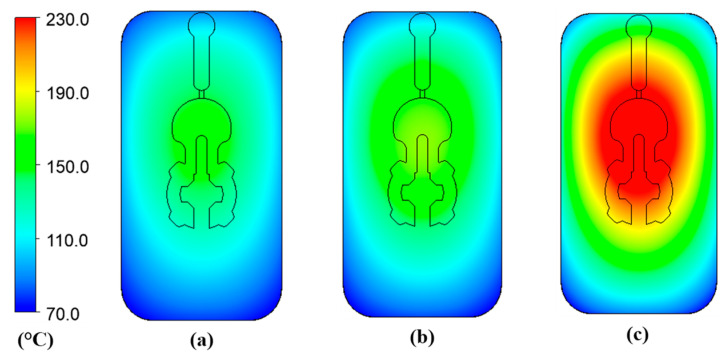
Temperature distribution of stamp with a gas temperature of (**a**) 200 °C, (**b**) 300 °C, and (**c**) 400 °C.

**Figure 15:**
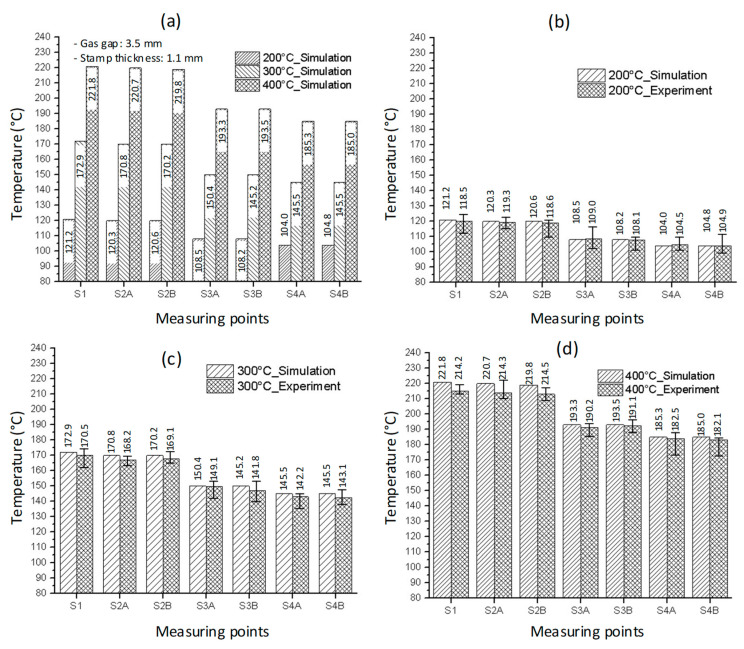
The comparison of temperatures (**a**) at measuring points with different inlet temperatures and comparison of simulation and experiment with the inlet temperature of (**b**) 200 °C, (**c**) 300 °C, and (**d**) 400 °C with a heating time of 20 s and stamp thickness of 1.1 mm.

The inlet temperature is a critical variable that has a significant impact on the heating process. As a result, this parameter was investigated in this study using the model shown in Figure 5, Figure 6, Figure 7 and Figure 8 with gas temperatures of 200 °C, 300 °C, and 400 °C; a product thickness of 0.2 mm corresponding to the stamp thickness of 1.1 mm; and a heating time of 20 s. Figure 14 illustrates the temperature distribution with a 1.1 mm stamp thickness at different air temperatures. This result demonstrates that the greater the inlet temperature, the more successful the heating process, resulting in a higher temperature at the center plate and a larger temperature difference on the plate. On the basis of the simulation results, the S1 temperatures were 121.2 °C, 172.9 °C, and 221.8 °C when the inlet gas temperatures were 200 °C, 300 °C, and 400 °C, respectively. When the 1.1 mm stamp thickness was used in this paper, the temperature distribution was better than in other cases after 20 s of heating, with a 3.5 mm gas gap. As shown in Figure 15a, a comparison was made between three different input temperatures. The experiment was repeated with the same gas gap to ensure that the simulation result was accurate. As shown in Figure 15b–d, the simulation and experiment were in good agreement.

### 4.3. Improvements to the Filling Process of the Micro-Molding Product Using the In-GMTC

**Figure 16:**
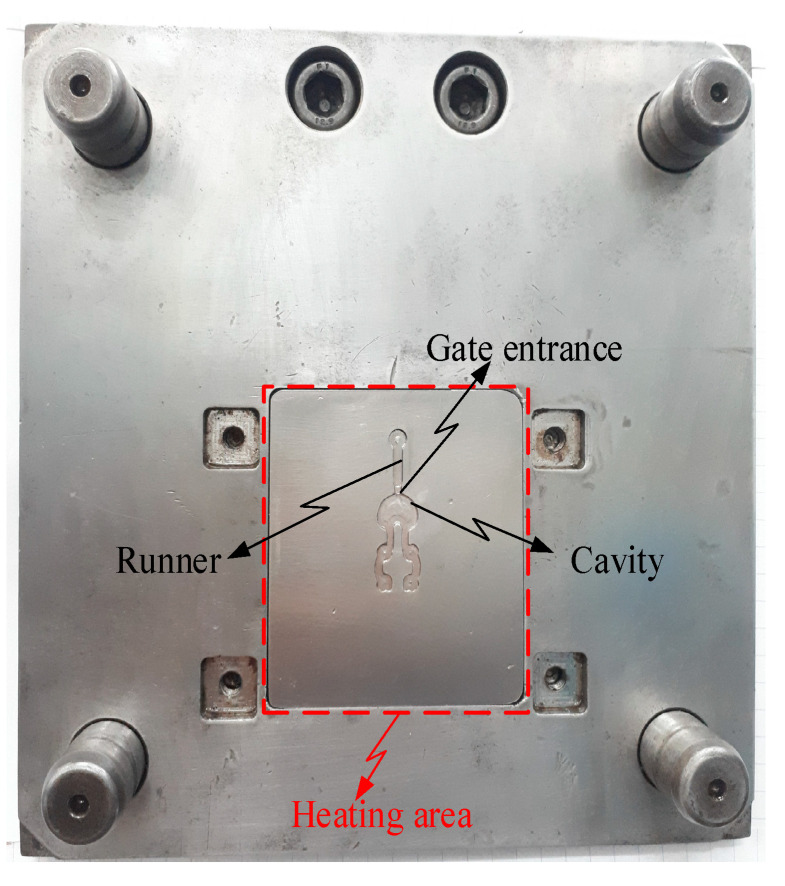
The mold for the front cover part with the gate heating area.

**Figure 17:**
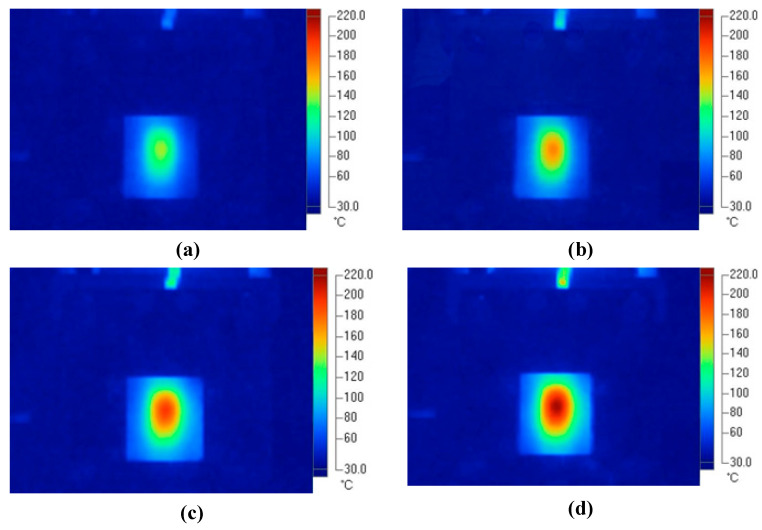
Temperature distribution of the core plate after 20 s of heating with gas temperatures of (**a**) 250 °C, (**b**) 300 °C, (**c**) 350 °C, and (**d**) 400 °C.

To verify the effectiveness of the In-GMTC in the injection molding cycle, a mold for a front panel product was used. The dimensions of this product are shown in Figure 5. The melting material was polyamide 6 + 30% glass fiber (PA6 + 30GF), and the product thickness was 0.2 mm, which made it a thin-walled product. In order to fully fill the cavities, the mold temperature needed to be set as high as possible, as compared with the appropriate mold temperatures of between 20 and 80 °C for common injection molding. This setting provides easy flow due to the reduction in the frozen flow layer. However, when the mold temperature is set at a high value, more energy is wasted, and other issues arise such as warpage and flashing. In order to address these issues, we propose the local mold temperature control presented in this paper. Instead of maintaining the entire mold plate at a high temperature, the temperature at the gate area was controlled at the local mold temperature via local air pre-heating at the start of the molding cycle. When the melt flow passes through the gate area, the high temperature reduces the pressure loss of the melt flow. The core plate, which includes the cavity and gate areas, is shown in Figure 16. The gate area, like the above structure, was redesigned with a steel insert to improve heating efficiency. The dimensions of this insert were 77.4 × 70 × 5 mm^3^. In this case, gas at temperatures of 200 °C, 250 °C, 300 °C, 350 °C, and 400 °C were used with a heating time of 20 s to observe the effect of the gas temperature on the filling process. An infrared camera was used to capture the temperature distribution at the end of the heating step in order to verify the heating efficiency and local heating. The real molding cycle was then performed using the parameters listed in Table 2. The molding cycle ran for 20 cycle products for each gas temperature case to reach system stability, and then the products from the next 10 cycles were collected to compare the melt flow length. For different gas temperatures, Figure 17 shows the temperature distribution and melt flow pattern. This result shows that the temperature at the cavity side clearly increased and the thermal energy was focused in the gate area, which reduced the pressure drop of the melt fluid and increased the filling ability of the molding cycle. In addition, because the high temperature only concentrates at the insert, the cooling step could be easily achieved due to this process requiring only a little thermal energy being removed from the insert, as opposed to thermal energy being removed from the entire volume of the mold plate as is the case in other heating methods, such as heater heating or steam heating.

**Figure 18:**
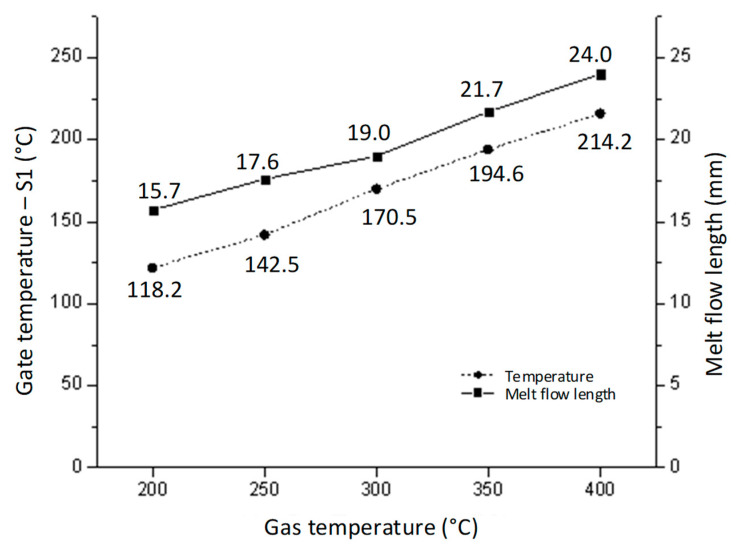
The melt flow length and gate temperature under different gas temperatures for the In-GMTC.

**Figure 19:**
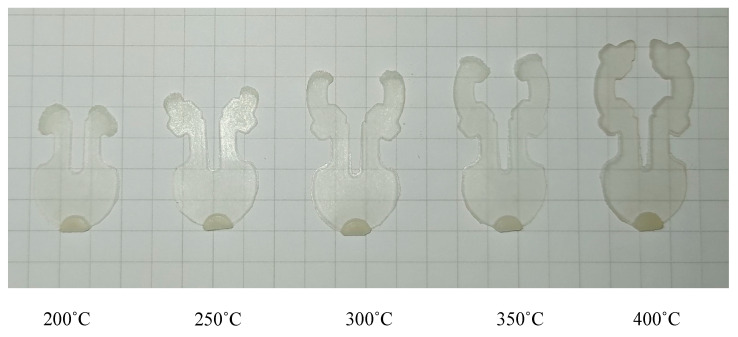
The melt flow length of front cover part after molding with the In-GMTC under different hot-gas temperatures.

Figure 18and Figure 19 show the gate temperatures at the S1 point and the filling percentage values that were measured. The temperature distribution shows that the high temperature was concentrated specifically in the gate area, which was heated in 20 s by the hot gas. This means that the mold plate was maintained at a low temperature throughout the molding cycle, resulting in less warpage and flashing and less energy wastage when compared to standard cases. When the gas temperature increased from 200 to 400 °C, the gate temperature rose from 118.5 to 214.2 °C with a 20 s heating time. In other studies, the filling process was more effective when the mold temperature was higher than the glass transition temperature. Figure 19 shows that when the gate temperature increased to over 216 °C, the cavity was fully filled. This demonstrates that the front cover part was able to fully fill in this mold design when the gas temperature was higher than 214.2 °C. Furthermore, these results show that the In-GMTC is highly efficient in term of improving the melt flow length for thin-wall injection molding products, as evidenced by the increase in the filling percentage from 65.4% to 100% (full fill) when the mold plate was maintained at approximately the same temperature.

## 5. Conclusions

In this study, the internal gas-assisted mold temperature control (In-GMTC) was established with different insert thicknesses (t) and gaps between the gas gate and heating surface (G) to achieve fast mold surface temperature control. Then, along with the local gate temperature control, the In-GMTC was used for the thin-wall injection molding cycle. The following conclusions were drawn from the results:

- The heating process was clearly affected by the stamp thickness. A thinner stamp provided a higher heating rate with a small heating area; however, a thicker stamp provided a better temperature distribution with a large heating area.
- Similarly, along with stamp thickness, the distance between the gas gate and the heating surface also affected the heating rate and the temperature distribution. The smaller this gap was, the more suitable the application for smaller heating areas with higher heating rates.
- The heating process using the In-GMTC was predicted fairly accurately using the ANSYS software with the CFX module.
- The use of the In-GMTC for a real molding cycle demonstrated that the melt flow length improved significantly when the In-GMTC was used at the melt gate. The temperature distribution at the core plate was determined using an infrared camera. Only the gate area was heated, while the other areas were maintained at the same temperature. In general, a complete cavity fill could be achieved with a gas temperature of 400° and a 20 s heating time.

## Figures and Tables

**Table 1:** Material properties (for simulation).

Material	Properties	Unit	Value
Air	Molecular mass	kg/kmol	28.96
	Density	kg/m^3^	1.185
	Specific heat capacity	J/kg °K	1004.4
	Dynamic viscosity	kg/ms	1.831 × 10^−5^
	Thermal conductivity	W/m°K	0.0261
Steel	Molecular mass	kg/kmol	55.85
	Density	kg/m^3^	7854
	Specific heat capacity	J/kg °K	434
	Thermal conductivity	W/m°K	60.5

**Table 2:** The molding parameters for the front cover plate product.

Molding Parameter	Unit	Value
Injection speed	cm^3^/s	33.0
Injection pressure	Bar	60.0
Injection time	s	1.0
Packing time	s	1.5
Packing pressure	Bar	35.0
Cooling time	s	15.0
Mold temperature	°C	50.0
Melt temperature	°C	265.0
Pre-heating time by In-GMTC	s	20.0

## Data Availability

The data used to support the findings of this study are available from the corresponding author upon request.
